# Inductive Frequency-Coded Sensor for Non-Destructive Structural Strain Monitoring of Composite Materials

**DOI:** 10.3390/s24206725

**Published:** 2024-10-19

**Authors:** Angelica Masi, Martina Falchi, Danilo Brizi, Eliana Canicattì, Guido Nenna, Agostino Monorchio

**Affiliations:** 1 Department of Information Engineering, University of Pisa, 56122 Pisa, Italy; danilo.brizi@unipi.it (D.B.); agostino.monorchio@unipi.it (A.M.); 2 Consorzio Nazionle Interuniversitario per le Telecomunicazioni (CNIT), 43124 Parma, Italy; guido.nenna@cnit.it; 3 Free Space s.r.l., 56121 Pisa, Italy; eliana.canicatti@free-space.it

**Keywords:** composite materials, deformation, spatial localization, radiofrequency, sensors, structural health monitoring

## Abstract

Structural composite materials have gained significant appeal because of their ability to be customized for specific mechanical qualities for various applications, including avionics, wind turbines, transportation, and medical equipment. Therefore, there is a growing demand for effective and non-invasive structural health monitoring (SHM) devices to supervise the integrity of materials. This work introduces a novel sensor design, consisting of three spiral resonators optimized to operate at distinct frequencies and excited by a feeding strip line, capable of performing non-destructive structural strain monitoring via frequency coding. The initial discussion focuses on the analytical modeling of the sensor, which is based on a circuital approach. A numerical test case is developed to operate across the frequency range of 100 to 400 MHz, selected to achieve a balance between penetration depth and the sensitivity of the system. The encouraging findings from electromagnetic full-wave simulations have been confirmed by experimental measurements conducted on printed circuit board (PCB) prototypes embedded in a fiberglass-based composite sample. The sensor shows exceptional sensitivity and cost-effectiveness, and may be easily integrated into composite layers due to its minimal cabling requirements and extremely small profile. The particular frequency-coded configuration enables the suggested sensor to accurately detect and distinguish various structural deformations based on their severity and location.

## 1. Introduction

Composites rapidly emerged as one of the most widespread and significant classes of engineering materials. The ability to tailor their mechanical and chemical characteristics according to the particular operating conditions allows the accomplishment of exotic combinations of stiffness, strength, toughness, lightness, and corrosion resistance [1]. Currently, a growing need for structural composite materials is pushing research in several industries such as aerospace, wind turbines, transportation, and medical equipment [2].

In more detail, a composite material can be defined as an artificial arrangement of two or more materials that exhibit, in their globality, enhanced qualities compared to the individual components separately used. Typically, one element of the composite serves as the matrix forming a continuous phase, while the other elements provide some kind of reinforcement. The final qualities of the material, particularly stiffness and strength for structural composites, are determined by the type, number and arrangement of the reinforcing [3]. Due to their countless declinations, different categories of composites have been proposed in the literature. A possible classification is based on their matrix constituent, which can be polymeric, metallic, or ceramic [4]. A further categorization considers the structural characteristics of the reinforcement. Specifically, fiber-reinforced [1,5,6,7,8], laminar [9,10], and particulate composites [11,12] can be distinguished.

**Figure 1:**
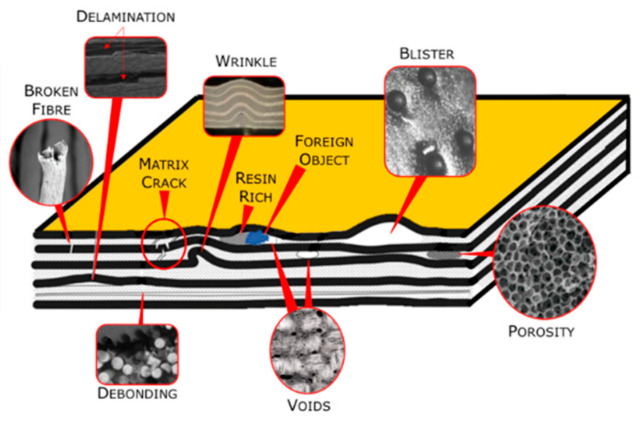
Illustration of the different kinds of defects that may occur in a composite [18].

As a matter of fact, monitoring the health status of composite materials is crucial since defects may appear during manufacturing or routine usage, leading to changes in mechanical characteristics, thus reducing performance and service life [13,14,15]. Mechanical impacts are the most common stresses that cause damage during normal operation [16]. Moreover, laminated composites often experience delamination, a significant type of failure caused by repetitive longitudinal stress and strain over time [17]. Figure 1 provides a comprehensive visual representation of the potential anomalies that can arise in a composite material [18].

In this context, structural health monitoring (SHM) systems, especially if they are able to combine non-destructive testing (NDT), are becoming extremely important to identify and quantify deterioration in composite materials. These techniques enhance safety, availability, and reliability, while also reducing maintenance costs [19]. SHM NDT systems include a variety of methodologies to evaluate the deformation and structural integrity of materials, both on their surface and internally, without causing any damage and not affecting their functionality [20]. The visual inspection and dye penetration techniques are the simplest NDT methods used to detect visible defects in composite materials. They are relatively easy to implement and cost-effective. However, they preclude a comprehensive analysis of the overall structure [21,22,23]. To face this issue, imaging techniques such as X- and γ-ray radiography are considered to be a valid approach [24,25]. This methodology provides an assessment of the health condition of a composite material at a specific moment, but does not have the capability for continuous monitoring over time, especially due to their negative side effects for human operators and high costs [24]. On the other hand, to accomplish a dynamic monitoring of the entire structure, the ultrasonic technique (UT) and infrared thermography (IRT) can be used [26,27,28,29,30]. The UT is mostly employed for fast and accurate detection of structural anomalies in sound conducting materials. Similarly, IRT allows for detecting infrared energy emissions from an object by quantifying and mapping thermal patterns, enabling a real-time and large-field inspection of the composite. Although these techniques find wide applicability, they are nevertheless characterized by high costs, complexity and requirements for significant time and computational resources [20]. Moreover, they exhibit a considerable susceptibility to external factors and inaccuracy when dealing with complex geometries [23]. In addition, the continuous temporal monitoring of the dynamic strains can also be accomplished through different instruments such as accelerometers, strain sensors and optical fibers [31,32]. These kinds of sensors have the advantages of geometric versatility, wide dynamic range and high sensitivity [33,34]. Conversely, they can only provide information from one single location, preventing a complete monitoring of the global structure. To tackle this limit, multiple sensors can be installed, but this results in a bulky solution that may have negative effects on the overall mechanical performance of large structures due to the elevated number of required connections [35].

In this context, electromagnetic fields can also be conveniently exploited. By interacting with the composite material, they can detect defects or deformations within the object [36,37]. Several microwaves and RF sensor technologies have been developed to perform no contact and real-time monitoring. However, their main limitation lies in finding a solution that combines high sensitivity, affordability, easy integration, and minimal cabling. High-sensitivity strain monitoring sensors adopting Split-Ring Resonators (SRRs) have been developed in recent studies [38,39]. However, in order to improve the quality factor and sensitivity of the system, a positive feedback loop, requiring the inclusion of batteries, is included. This makes the system quite complex and challenging to be integrated into composites. On the other hand, passive solutions have also been proposed in the literature [40,41,42,43]. Despite their effectiveness and easy integration within composite materials, their main drawback is the localized and punctual analysis, which requires the installation of additional monitoring equipment to cover a larger area. A multi-sensor radiative system was designed in [44] to cover a broader investigation area, minimizing wiring requirements. However, due to the poor-quality factor of the resonating sensing elements, the solution exhibits reduced sensitivity. Furthermore, a recent study outlined the development of a highly sensitive sensor composed of interconnected split-box resonators, thus offering an uninterrupted detection of cracks [45]. However, the system’s operational frequency band does not guarantee significant penetration depths.

In order to improve the current state-of-the-art electromagnetic non-destructive structural health monitoring (NDT SHM) sensing devices, this manuscript proposes the design of a novel RF sensor to examinate various deformation conditions within a composite material, thus helping to prevent failure caused by overloading. Specifically, the design of this sensing system consists of a feeding copper line, requiring only one connectorization and inductively coupled to a series of frequency-coded spiral resonators (SRs). In addition, the spatial localization of the stress can be accomplished by the frequency-coded SR arrangement. Finally, this sensing technology possesses the capability to accurately detect and identify multiple structural deformations simultaneously, making it an excellent option for non-destructive SHM. The SRs’ configuration exhibits operating frequencies within the range of 100–400 MHz, resulting in a balance between the penetration depth and sensitivity of the radiative system. By exploiting PCB technology, the proposed detection system also offers a cost-effective and easily integrable solution, especially within plies of laminated composites. The model may be customized according to the application by adding more spiral resonators and adjusting power parameters.

The remainder of the manuscript is organized as follows. Section 2 presents the analytical model that explains the operating principle of the sensing system, while Section 3 details the numerical design and characterization of the PCB prototype. Section 4 provides a description of the numerical and experimental scenarios, as well as a discussion of the obtained results derived from the analysis of composite deformation. Finally, Section 5 discusses the conclusion and outlines future works.

## 2. Analytical Model

**Figure 2:**
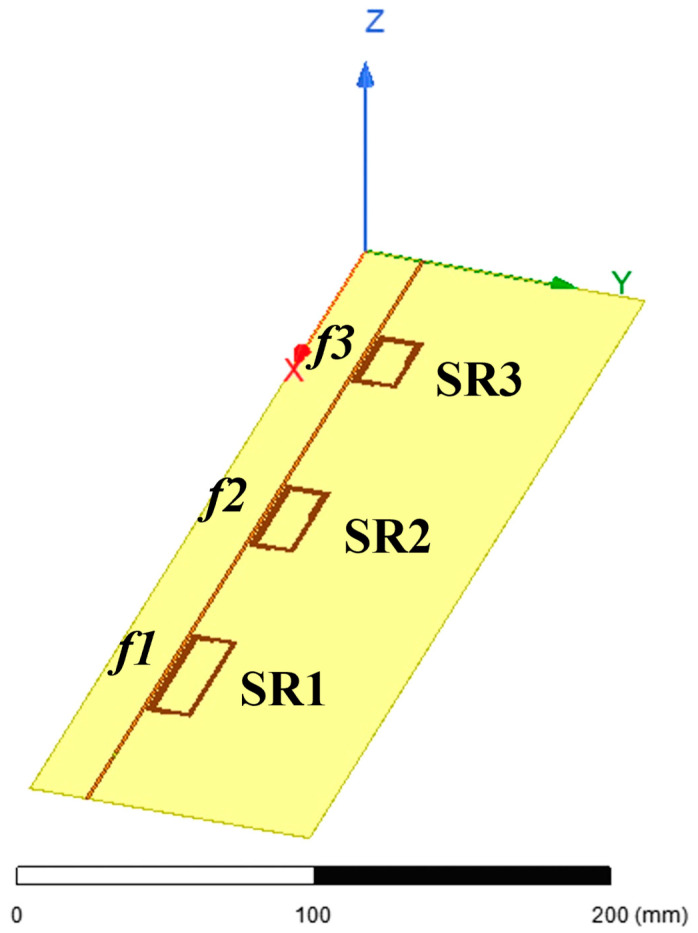
Perspective view of the proposed radio frequency sensing system 3D CAD.

Figure 2 depicts the sensing system configuration proposed in this paper. It consists of an actively fed strip line that is inductively coupled with three spiral resonators (SRs). Each SR is designed to self-resonate at its own resonance frequency, which is a result of its intrinsic inductive behavior and parasitic capacitance. This design avoids any requirement for external lumped reactive elements and creates a frequency-coded spatial localization [46]. More precisely, the sensing structure is placed upon or within a composite material and the resonant SRs enable the identification of the abnormalities due to the various stress conditions. The fed strip line serves the purpose of both giving power to the SRs and collecting back the signal variations. Indeed, by measuring the input impedance of the line, it is possible to detect the changes in the three SRs’ resonances, including amplitude and frequency variations (control parameters). As extensively explained in [47], the resonators have smaller dimensions compared to the operating wavelength. Therefore, at the resonant frequency, each SR produces a substantial electric field in the gap between the turns, thus becoming extremely sensitive to variations in the dielectric characteristics of the surrounding medium [48,49]. These changes are a consequence of the deformation presence within the composite material. It is important to notice that the frequency-coded SRs also allow a spatial localization of the stress, since each resonant frequency is associated with a precise SR position along the fed strip line.

Several studies were conducted in the literature in order to define the analytical model of passive SRs and their interactions with external RF elements [50,51,52,53,54]. Typically, if the electrical size is significantly small with respect to the applied wavelength, the planar SR can be represented by an RLC series resonator as an equivalent circuit. The lumped parameters can be accurately determined using the extraction procedure described in [52]. This process offers a reliable strategy for predicting both the resonance frequency and its Q-factor. As a matter of fact, the SR-equivalent lumped elements depend on their geometry as well as on the properties of the surrounding medium. 

The straight copper strip line, inductively coupled with the SRs, is fed by a coaxial cable and it is modeled as an inductance connected in series with a resistance, as reasonable at those relatively low frequencies (hundreds of MHz) [55]. Hence, the neighboring passive SRs are activated via inductive coupling, in accordance with Faraday’s law. Essentially, the current flowing through the feeding line creates a magnetic field that induces a secondary current on the passive resonator due to the concatenated flux. As a result, a wireless transfer of energy between each SR and the active strip line is occurring [27]. Hence, by employing a single electrical connection to the power source, the overall sensing system can be simply integrated either on the surface or within the plies of a composite material. Moreover, the independent coupling of each SR with the active line allows the integration of a customizable number of SRs to properly monitor the desired area by exploiting the frequency coding. This independence among SRs can be guaranteed by leaving a sufficient distance between each pair of them; thus, their reciprocal mutual coupling can be neglected.

**Figure 3:**
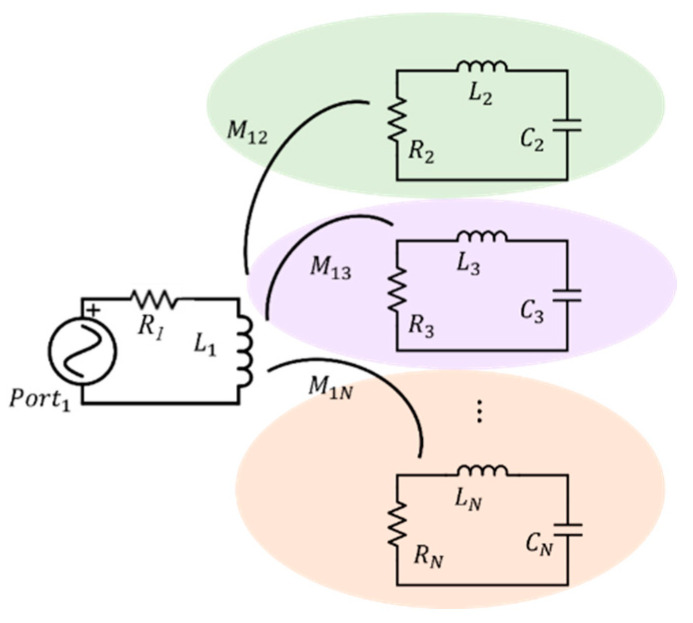
Schematic representation of the equivalent lumped circuit for *N* independent SRs inductively coupled with the active strip line.

Figure 3 illustrates the equivalent circuit of a generic sensing system composed of (*N* − 1) independent SRs [54]. In this model, $R_{1}$ and $L_{1}$ represent the resistance and inductance of the fed copper line, whereas each of the (*N* − 1) SRs is represented by a series circuit consisting of $R_{i}$, $L_{i}$ and $C_{i}$, for i=2,…N. The coupling coefficient *M*_1*i*_ accounts for the mutual coupling between the *i*-th SR and the strip line. By adopting the frequency domain, it is possible to express the Kirchhoff’s equations as in the following, based on the voltage of the feed line $V_{1}$:

(1) $$\begin{array}{c} \left\{\begin{array}{c} \begin{array}{c} {Z_{11}I_{1}+Z}_{12}I_{2}+\ldots +Z_{1N}I_{N}=V_{1}\\ Z_{21}I_{1}+Z_{22}I_{2}=0 \end{array}\\ \begin{array}{c} \vdots \\ Z_{N1}I_{1}+Z_{NN}I_{N}=0 \end{array} \end{array}\right. \end{array}$$

In this formulation, $Z_{11}$ represents the impedance of the strip line. $Z_{ii}$ and $Z_{i1}$, on the other hand, respectively, denote the self-impedance of the *i*-th SR and the coupling term between the *i*-th SR and the strip line. Thus, by elaborating (1) through simple algebraic manipulations, the fed strip line input impedance can be written as:

$$Z_{input}\left(\omega \right)=\frac{V_{1}}{I_{1}}=Z_{11}-\sum_{i=2}^{N}\frac{{\left(Z_{i1}\right)}^{2}}{Z_{ii}},$$

(2) $$\begin{array}{c} \frac{{\left(Z_{i1}\right)}^{2}}{Z_{ii}}=\frac{-\omega^{2}{M_{1i}}^{2}}{R_{i}+j\omega L_{i}+\frac{1}{j\omega C_{i}}} \end{array}$$

To obtain an estimate for the RLC parameters of each spiral, a de-embedding approach exploiting the model described in [52] can be applied. By elaborating on Equation (2), the resistance value of the *i*-th SR can be evaluated as the real part of the quantity reported in the next equation, Equation (3). Conversely, the SR inductance can be computed as the half derivative of the corresponding imaginary component when evaluated at the resonant frequency (see Equation (4)). Finally, the capacitance value can be obtained by using the resonance frequency relationship for an SR (as in Equation (5)).

(3) $$\begin{array}{c} R_{i}=ℜ\left\{\frac{{{\omega_{r}}_{i}}^{2}{M_{1i}}^{2}}{Z_{input}\left({\omega_{r}}_{i}\right)-Z_{11}\left({\omega_{r}}_{i}\right)}\right\} \end{array}$$

(4) $$\begin{array}{c} L_{i}=\frac{1}{2}\frac{\partial }{\partial \omega }\left\{I\left\{\frac{{{\omega_{r}}_{i}}^{2}{M_{1i}}^{2}}{Z_{input}\left({\omega_{r}}_{i}\right)-Z_{11}\left({\omega_{r}}_{i}\right)}\right\}\right\} \end{array}$$

(5) $$\begin{array}{c} C_{i}=\frac{1}{{{\omega_{r}}_{i}}^{2}L_{i}} \end{array}$$

Clearly, the above reported de-embedding procedure is valid in the hypothesis of negligible mutual coupling between each SR pair, as previously described. Since the SRs are resonating at different and well-separated frequencies, this hypothesis is effective.

The suggested sensing system detects deformation states within a material by measuring the change in the SRs’ resonant frequency and in the corresponding peak amplitude by collecting the fed strip line input impedance. When any of these conditions occur, the morphological characteristics of the material surrounding the sensor change, causing variations in the overall dielectric properties (${\varepsilon_{r}}^{\prime }$ and ${\varepsilon_{r}}^{\prime \prime }$) and affecting the SRs’ response. Specifically, when a force is applied on the composite structure, it creates stress (σ) through its cross-sectional area *A* ($\sigma =\frac{F}{A}$). Thus, a deformation ε takes place, based on Young’s modulus *E* ($\varepsilon =\frac{\sigma }{E}$). These changes in the mechanical status of the radiating system surrounding the medium can be characterized in terms of equivalent circuit (2). Thus, an analytical connection between the circuit’s lumped parameters and the variations in the material complex dielectric permittivity ${\varepsilon_{r}}^{\prime }$ and ${\varepsilon_{r}}^{\prime \prime }$ can be established [52]. In particular, the complex dielectric permittivity directly affects the SR parasitic capacitance term $C_{i}$ in Equation (2), hence significantly influencing the sensor response [56].

## 3. Design Procedure

### 3.1. Numerical Design of the Optimal SRs

The numerical design was carried out by exploiting a full wave simulator (Ansys® Electronics, 2022 R1). As previously mentioned, the conceived SHM system consists of three multi-turn planar spiral resonators inductively coupled with an actively fed straight strip line. These components are positioned on a 100 mm × 360 mm I-Tera dielectric substrate (ISOLA Group, Chandler, AZ, USA) presenting a thickness of 0.09 mm (ε_r_ = 3.45, tanδ = 0.003).

As Figure 2 illustrates, the SRs are positioned with their centers 9 mm away from the actively fed straight line. The line is fed with a port positioned toward the end, at *x* = 330 mm with respect to Figure 2. To achieve an acceptable sensitivity level, the geometry of each SR was optimized by maximizing the quality factor parameter. This quantity is formally defined as the ratio of the total energy stored by the resonator against the energy lost per cycle (*Q = E*/∆*E*) [57]. Basically, this implies that when the Q-factor increases, the stored reactive energy has a more significant impact compared to the losses. Since the detection of structural irregularities within materials is mainly dependent on the stored energy perturbation, it can be deduced that an optimized Q-factor results in a more responsive system.

To satisfy the requirement for a highly sensitive monitoring device, the first step of the optimization procedure is to maximize the overall area covered by each SR, compatible with the fed strip line length, the desired spatial resolution and the reciprocal SRs’ independence. These last conditions are arbitrary and depend on the specific application. In this case, the outer dimensions of the longer sides of the SRs, reported from the first to the last resonator (Figure 2), have been selected as 50.45 mm, 40.45 mm, and 30.45 mm, respectively. In addition, the distance between two adjacent SRs’ centers is 105 mm, i.e., at least double of the resonators’ size. These dimensions allow us to also consider the negligible the mutual coupling between each couple of resonators, necessary to achieve an independent operation modality. It must be noticed that the shorter side length of all the three resonators is maintained at 15.45 mm, since it is sufficient to concatenate the major part of the magnetic field produced by the fed straight line.

After determining the external dimensions of the SRs, we proceeded with the design of the spiral geometry to ensure that they operate within the selected band of 100–400 MHz. These frequencies ensure both the accomplishment of high Q-factors and a satisfactory level of miniaturization for the resonators.

**Figure 4:**
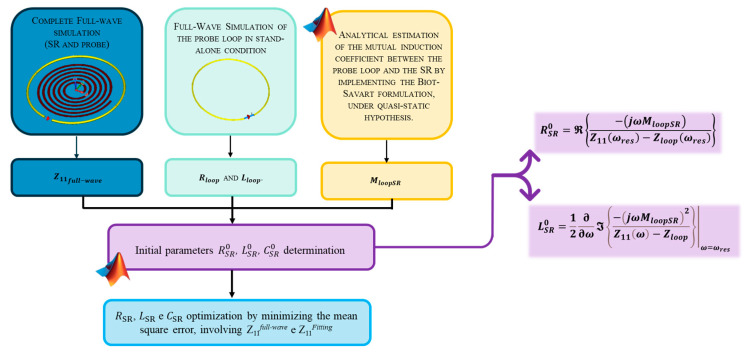
Flowchart summarizing the main steps for extracting the RLC parameters of a general SR sensing element.

The Q-factor can be determined by exploiting the RLC equivalent circuit for a spiral resonant element, as extensively discussed in the literature [52,58,59,60]. The expression is as follows:

(6) $$Q_{F}={\left.\frac{\omega L}{R}\right|}_{\omega =\omega_{0}}$$

with $\omega_{0}$ representing the resonant frequency. Several studies have been conducted in the literature to investigate the impact of the resonator geometric parameters on its performance. As a result, the number of turns, strip width, thickness and gap between consecutive turns have been demonstrated to be the most influential characteristics [61,62]. Consequently, we employed the methodology outlined in reference [52] to identify the configuration with the highest Q-factor. The flowchart in Figure 4 summarizes the major steps in the RLC parameters’ extraction procedure, identifying the optimal geometry of the sensor. The system components were designed by using a PCB conventional 35 µm thickness for the lossy copper microstrip. Indeed, as demonstrated in the literature [46], the quality factor of this resonant structure is unaffected by the microstrip’s thickness. Thus, by implementing the optimization procedure and applying the quality factor formulation (6), we successfully achieved the design of the three spiral resonators.

**Figure 5:**
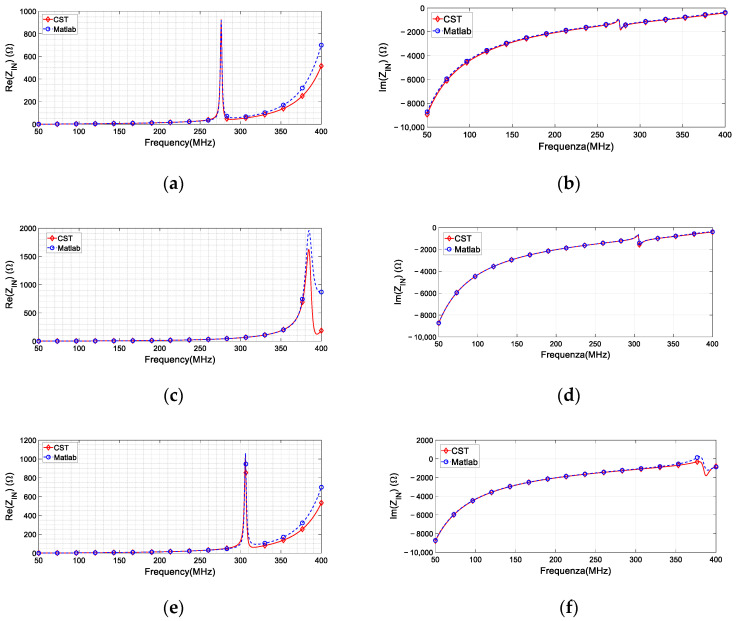
Comparison between simulated Z_input_ (solid red line) and Z_input_ as reconstructed from the RLC parameter estimations (fitting, dashed blue line). (**a**) Real part with the presence of only SR_1_. (**c**) Real part with the presence of only SR_2_. (**e**) Real part with the presence of only SR_3._ (**b**) Imaginary part with the presence of only SR_1_. (**d**) Imaginary part with the presence of only SR_2_. (**f**) Imaginary part with the presence of only SR_3_.

The optimal SR_1_ design was determined to be a multi-turn structure with 2.5 windings and a pitch of 0.9 mm. SR_2_ and SR_3_ were created with the same pitch, but a different number of turns, specifically 2.75. The obtained optimized Q-factor approximately measures 115 for SR_1_, 113 for SR_2_ and 37.5 for SR_3_. The corresponding self-standing resonant frequencies are 269, 283, and 359 MHz. Table 1 summarizes the geometrical and electrical characteristics of all the proposed designs for the spiral resonators. Conversely, Figure 5 illustrates a comparison between the curves obtained using the circuital parameter extraction technique and the impedance behavior derived from full-wave simulations. Specifically, in Figure 5a,c,e, the real part of the straight fed line input impedance has been evaluated for each SR singularly present. Similarly, Figure 5b,d,f reports the behavior of the imaginary part.

**Figure 6:**
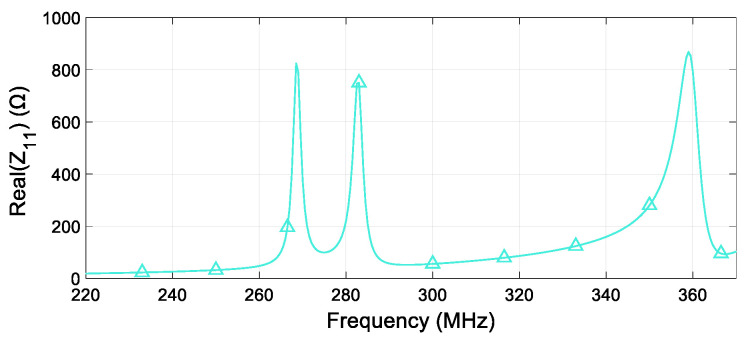
Real part of the fed strip line input impedance as a function of the frequency in the unloaded condition. The operating frequencies of the three system unit cells are approximately 269, 283, and 359 MHz, respectively, for SR1, SR2 and SR3.

After identifying the final sensing shape, we evaluated the overall system response in the unloaded condition. This means that we numerically tested the system without placing any specimen near the sensing device. By analyzing the active line input impedance behavior in the real component, as reported in Figure 6, three distinct resonances at the desired SRs resonant frequencies can be spotted. Therefore, the independence of the SRs is also guaranteed, as requested. This analysis provides evidence of the overall design process’s reliability.

### 3.2. Prototype Fabrication

To create an accurate, mechanically robust, and extremely flexible prototype (easy to be integrated inside a composite material), the overall system was manufactured by adopting Printed Circuit Board (PCB) technology.

**Figure 7:**
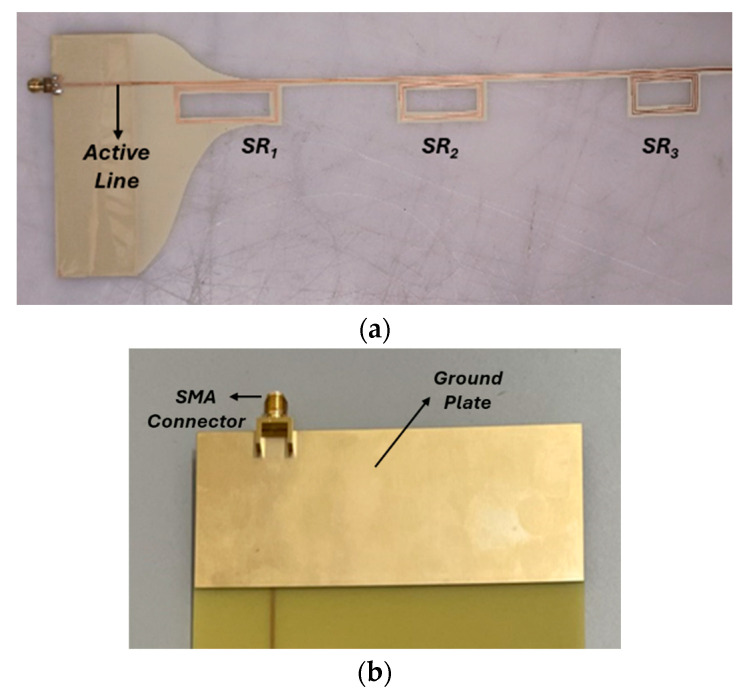
Photos of the experimental prototype. (**a**) Top view of the radiative sensing system PCB. The substrate without the copper strip was removed to allow an easier integration into the composite material. (**b**) Bottom view of the PCB of the radiative sensing system; the large copper ground, created to guarantee a solid connection with the VNA, can be appreciated.

We followed all the fabrication details reported in Section 3.1. In particular, the chosen dielectric substrate was I-Tera MT40 ($\varepsilon_{r}$ = 3.45, tanδ = 0.003) with a thickness of 90 µm, above which 35 µm thick copper strips were etched. The mechanical properties of the selected substrate provide high flexibility and improved capability to conform to various deformation states of the material under analysis. We slightly modified the original CAD geometry to enable an easier feeding connection, since this is one of the most delicate aspects for the correct operation of the prototype. To accomplish this objective, we expanded the ground area at the back of the substrate with a rectangular metal region 35 µm thick, as illustrated in Figure 7a,b. Thanks to this expedient, the connector ground can be soldered upon a larger copper area, thus ensuring robustness and reduced measurement noise. After that, we connected the sensing system to a VNA (VNA P9374A, 300 kHz–20 GHz, Keysight, Santa Rosa, CA, USA) by using a SMA PCB connector.

Finally, the dielectric substrate not covered with the copper strip was removed to allow a better integration within a composite material. Indeed, the liquid resin of a typical fiberglass composite can easily permeate the hollow parts of the prototype, fixing it strongly inside the structure during the curing process, thus guaranteeing a highly sensitive measurement in terms of stress and strain recording.

## 4. Deformation Analysis

### 4.1. Numerical Set-Up and Results

**Figure 8:**
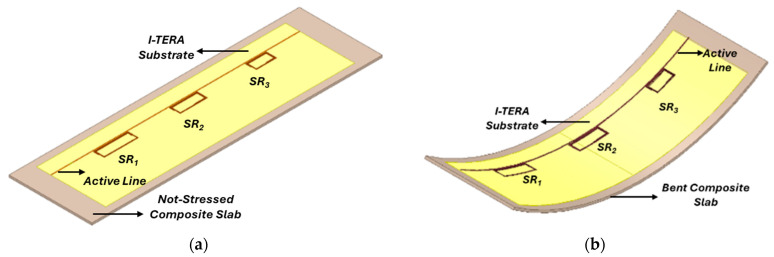
The two simulated scenarios were conceived to evaluate the sensing performance in case of deformation. (**a**) The set-up in the absence of applied forces (baseline case); (**b**) the structure subject to bending stress.

To evaluate the capability of the proposed radiative system to monitor and quantify different deformation states, a comprehensive numerical analysis was conducted by again exploiting the electromagnetic solver Ansys Electronics (HFSS). In particular, we included the aforementioned sensing arrangement over the surface of a rectangular slab that represents the composite material under analysis (refer to Figure 8a). The slab has external dimensions of 120 mm × 420 mm with a thickness of 3 mm. The complex dielectric permittivity of the material was chosen as $\varepsilon_{r}=$ 4.3 and tanδ=0.021. This represents a material with the common properties of fiberglass composites [63].

In order to evaluate the effect of the material deformation on the sensor output (i.e., the input impedance of the fed straight strip line), a second CAD scenario involving a bending was simulated, as shown in Figure 8b. The specimen under testing underwent a bending with a curvature radius of 500 mm. It is worth noticing that the sensing system positioning on the external surface of the composite specimen was motivated by considering that the stresses are most significant on that part.

**Figure 9:**
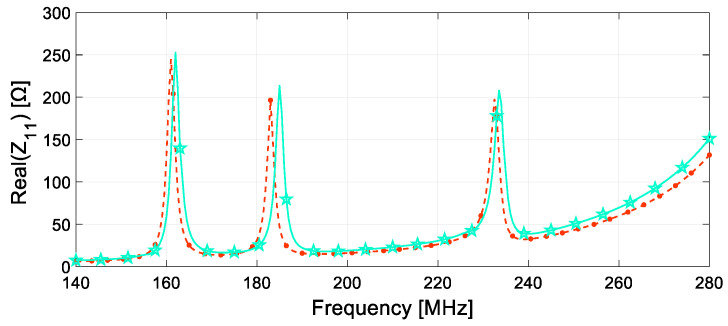
Numerically evaluated system response obtained from the investigated scenarios. The real part of the system input impedance as a function of the frequency for the unloaded (red, dashed curve) and bent slab (green, full curve) are represented. The operating frequencies of the three system unit cells under the unloaded condition are approximately 161, 183, and 232 MHz, respectively, from SR1 to SR3. Due to bending stress, the operating frequencies of the three unit cells shift approximately to 162, 185, and 233.5 MHz, respectively, from SR1 to SR3, in conjunction with a significant variation in peak amplitude.

As explained in the Section 2, the resonant SRs are highly sensitive to changes in the dielectric properties of the surrounding medium, enabling the identification of abnormalities resulting from various stress conditions. By analyzing the input impedance, amplitude and frequency shifts in the SRs’ resonances can be detected, revealing deformation within the composite material. Figure 9 illustrates the sensor’s response for both the analyzed scenarios. Specifically, the red, dashed curve depicts the system’s output when no load affects the slab, which serves as the reference baseline. The operating frequencies of the three system unit cells, from SR_1_ to SR_3_, can be determined by analyzing the real component of the input impedance. These frequencies are roughly 161, 183, and 232 MHz, respectively. The values are lower than what was reported in the design section; indeed, the presence of the dielectric slab representing the composite adds a capacitive content to the SRs, reducing the corresponding resonance frequency.

Conversely, in the second case study, the sensor response was evaluated in relation to deformation, and the behavior is illustrated by the green, full curve in Figure 9. Essentially, we noticed a slight upshift in the SRs’ resonating frequency and a corresponding increase in amplitude compared to the baseline condition. The operating frequencies for this second arrangement, starting from SR_1_ to SR_3_, are equal to 162, 185, and 233.5 MHz. These values represent a percentage increase of approximately 1%, 1.1% and 0.9% compared to the reference case. In addition, a much more significant variation is appreciable in the peak amplitudes of the real part of the input impedance, rising by 3.2%, 8%, and 4.9% in comparison to the original scenario. 

The observed behavior is coherent with the physics, as the capacitance and inductance of the spiral resonators are affected by geometry variations due to the state of deformation, thus confirming the validity of the proposed sensing configuration [64,65].

### 4.2. Experimental Set-Up and Results

**Figure 10:**
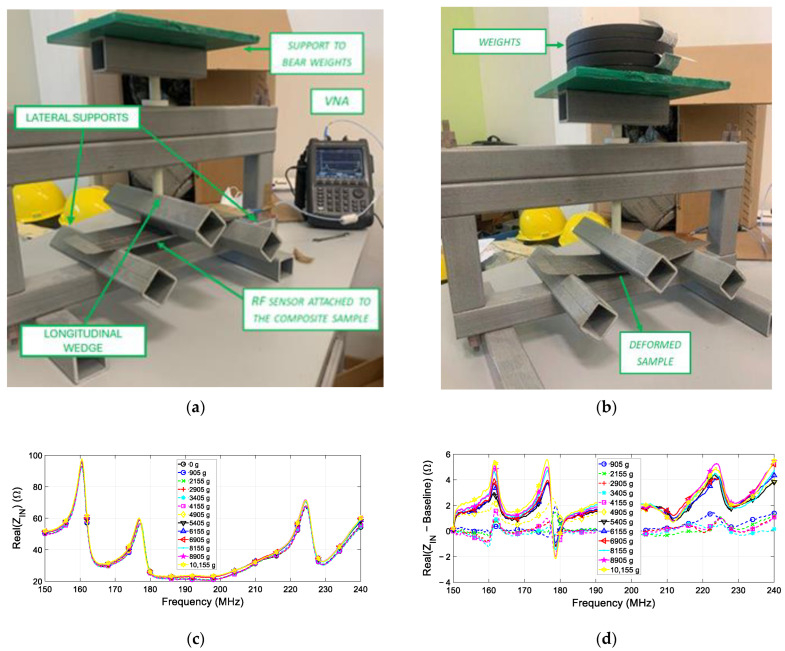
(**a**) Experimental set-up in the rest condition, including the polymeric bending machine and the composite specimen under testing (positioned between the three support wedges and integrating the sensor). (**b**) Experimental set-up under the stress condition, where the slab is bent due to the application of vertical weight. (**c**) Real part of the sensor’s fed line input impedance as a function of frequency for different applied weights (reported in the legend). The baseline case is represented by the black, circled line. (**d**) Curves were obtained by subtracting the baseline from the real part of the impedance, as a function of frequency, for different applied weights (reported in legend).

Once the proposed technology was validated via the analytical model and numerical simulations, we carried out experimental tests to assess the sensing performance of our prototype in a more realistic scenario. In particular, the sensor PCB prototype shown in Figure 7 was integrated within the plies of a 3 mm thick fiberglass composite with the same external dimension of the numerical model (Figure 10a,b). Notably, the sensor is positioned right below the first ply, to be most sensitive to deformation, as previously discussed.

Then, to conduct a bending test with controlled and repeatable experimental conditions, we realized a plastic structure with a sturdy base, specifically engineered to provide support for the specimen under testing (Figure 10a,b). The upper part of the structure is responsible for transmitting the load from the top to the sample through a longitudinal wedge, in turn connected to the movable crossbar of the machine. In essence, the test consists of an indirect deformation measurement, accomplished by applying a gradual and continuous concentrated load perpendicular to the longitudinal axis of the sample under investigation. This results in the generation of a bending moment on the two lateral machine-holding supports, allowing an immediate evaluation of the applied stress impact on the resonance of the various sensing resonant elements. Finally, the impedance measurements were performed by connecting the sensor’s SMA connector to the VNA.

In order to exclude the electrical loading effect of the plastic structure on the sensor’s response, the reference measurement (“no load” scenario) was acquired while the three wedges were just in contact with the sample under rest conditions (see Figure 10a). In particular, the overall good agreement with the numerical results can be observed. Subsequently, weights of known magnitudes (specifically, 905 g, 2155 g, 2905 g, 3405 g, 4905 g, 5405 g, 6155 g, 6905 g, 8155 g and 10,155 g) were placed on the upper support. This led to a gradual deformation of the specimen, as illustrated in Figure 10b. Following the same procedure as in the numerical results section, the raw experimental measurements of the sensor’s input real impedance are presented in Figure 10c. To highlight the variations produced on the local maxima in correspondence with each spiral resonance frequency by the increasing loading process, the baseline was subtracted from each loading curve, as reported in Figure 10d.

From the graph, it is clear that a recurring trend in the real part of the sensor’s input impedance can be observed. Indeed, the SRs’ resonant peaks reach higher values as the applied weights increase. This behavior aligns with the theoretical modelization and with the results obtained from the full-wave simulations. Indeed, the different stress condition leads to a progressive increase in the amplitude of the input impedance real part, with a progressively more pronounced variation in correspondence to a more significative weight condition. Moreover, the stress applied in the experiment does not produce a substantial shift in resonance frequencies, and therefore they remain nearly constant for all the three SRs. It is worth noting that the chosen weights cause elastic deformation in the composite without inducing permanent damage, which is crucial for detecting excessive stress before it leads to failure.

**Figure 11:**
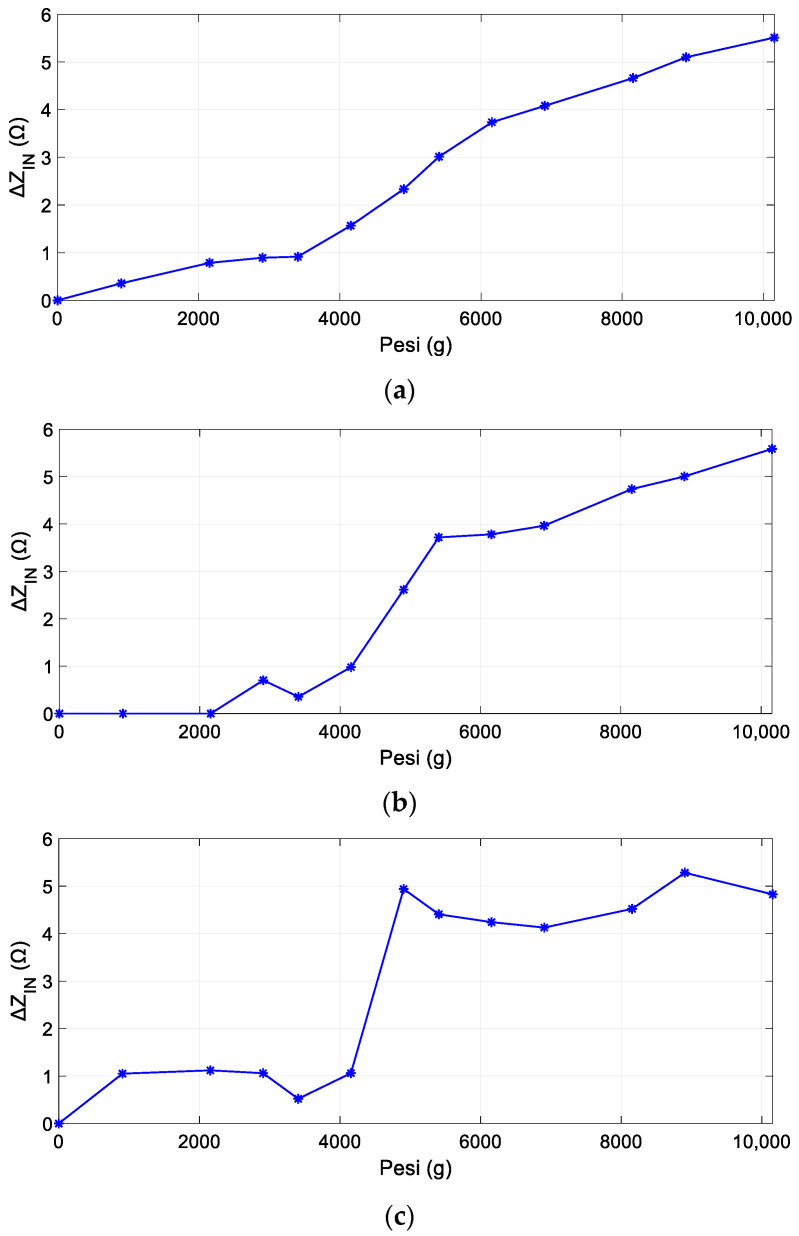
Variation of the sensor’s input impedance (maximum value of the real component) with respect to the baseline, as a function of the applied weights; (**a**) the first SR peak trend; (**b**) the second SR peak trend; (**c**) the third SR peak trend.

Figure 11 reports the variations in the resonating peaks of the sensor input impedance for various applied weights with respect to the baseline, always in the real component. These calculations were performed for each SR. Specifically, we can observe a consistent increase in the amplitude of the three peaks when the weights are increased. Hence, these findings confirm the effectiveness of the proposed technology in detecting deformation in the three specific regions where the sensors are placed, thereby ensuring the capability to precisely locate and quantify the deformation within the sample.

To summarize and have a better insight into the performance of RF sensors for non-destructive sensing, a comparison between the solution described in this work and the state-of-the-art structural health monitoring sensors is reported in Table 2. Compared to the other SHM RF technologies described in the literature, our sensor offers significant advantages, including superior sensitivity due to the Q-factor maximization, high customizability with the possibility to integrate an arbitrary number of resonant elements while requiring only a single connector, and cost-effectiveness. Additionally, it provides precise spatial localization thanks to the adopted frequency-coded approach and achieves a high penetration depth through the SRs’ resonance frequency optimization, in order to ensure both high sensitivity and the ability to investigate at large depths.

## 5. Conclusions

In this paper, a novel radiofrequency sensor to be integrated in composite materials for non-destructive structural strain monitoring was investigated. The suggested hardware system comprises an actively fed copper-line, inductively coupled with three self-resonant spiral resonators. The SRs have been optimized in order to maximize the Q-Factor and, thus, the system sensitivity. This structure allows for detecting changes in the stress status of a composite material by exploiting the variations on both amplitude and frequency of the respective resonant frequency. Thus, while the SRs enable the identification of abnormalities in the structure of the material, the strip line serves the purpose of both powering the device and collecting signal variations in terms of input impedance measurements. Full-wave simulations carried out on the conceived test case demonstrated excellent sensing performance, confirmed by the experimental measurements performed by positioning the fabricated prototype in contact with a 3 mm thick laminate. Therefore, the proposed technology offers a promising solution for non-destructive testing and evaluation of composite materials, addressing the critical need for reliable structural health monitoring in various industrial sectors. Certainly, future developments will focus on improving the proposed system to enhance detection range, thereby enabling the integration of the sensor into laminates with larger thicknesses than those addressed in this preliminary study. It is noteworthy that, although in this work our sensing solution was directed to provide a real-time monitoring of a progressive deformation status, the potential to detect defects internal to the composite material can be envisioned as well. In this context, incorporating advanced AI algorithms into our sensor system is a promising direction for future research, enhancing the sensor’s capability to both detect the presence and classify the nature of defects. AI algorithms, such as machine learning [71,72] and pattern recognition techniques [73], can be trained on a dataset of known defects in order to analyze the sensor data in real-time and classify defects based on their distinctive impedance patterns.

Clearly, additional work must be conducted to estimate the mechanical characteristics of engineering materials in order to establish a direct correlation between the induced strain and the sensor outcomes. In addition, future efforts will be directed towards advancing this technology to enable wireless operation. This enhancement will significantly broaden the applicability and convenience of the sensing mechanism, facilitating more versatile and non-invasive deployment across a range of real-world environments.

## Figures and Tables

**Table 1:** SRs’ geometric parameters.

Parameters	SR_1_	SR_2_	SR_3_
Longer side length	50.45 mm	40.45 mm	30.45 mm
Shorter side length	15.45 mm	15.45 mm	15.45 mm
Number of turns	2.5	2.75	2.75
Strip width	0.45 mm	0.45 mm	0.45 mm
Strip thickness	35 µm	35 µm	35 µm
Strip spacing	0.45 mm	0.45 mm	0.45 mm
Q-Factor	115.34	113.01	37.50
Resonant Frequency	269 MHz	283 MHz	359 MHz

**Table 2:** State-of-the-art comparison of radiofrequency and microwave sensors for non-destructive structural monitoring of metal/composite materials.

Ref	Sensor Structure	Operating Frequency	Investigated Material	Detection Method	Advantages	Disadvantages	Application Scenarios
[66]	Quarter-Wavelength Patch Antenna with Coaxial Feed	2.4 GHz	Metal	Changes in resonant frequency and impedance	Miniaturized, sensitive	Substrate material affects performance, deterioration of mechanical deformation	Deformation Monitoring
[67]	Chip-based UHF RFID	915 MHz	Metal	Variations in antenna impedance due to cracks	Wireless, passive, can detect fine cracks	Limited to specific materials, environmental sensitivity	Surface Crack Detection
[63]	Chipless RFID	2.45 GHz	Metal	Electromagnetic backscatter changes due to cracks	Wireless, passive, cost-effective	Limited to conductive materials	Surface Crack Detection
[68]	Frequency-Selective Surface (FSS) Sensors	10.2 GHz	Composite Materials	Changes in resonant frequency due to cracks or deformation	Can monitor large areas, sensitive	Complex design, may need precise alignment	Strain Sensing and Crack Detection
[69]	Coaxial Cable Sensor	1–10 GHz	Composite Materials	ETDR (Electrical Time Domain Reflectometry)	High sensitivity, real-time monitoring	Requires complex setup, potentially high cost	Crack Detection
[70]	Patch antenna fed by Microstrip lines	12 GHz	Metal	Changes in resonant frequency and impedance due to deformation	Accurate, miniaturized	Affected by substrate material, environmental conditions	Deformation Monitoring
T.W.	SRs Array Sensor	269–359 MHz	Composite Materials	Changes in impedance amplitude due to deformation	Sensitive, customizable (to extend the investigation area) while requiring only one connectorization, cost-effective, spatial localization, high penetration depth	Environmental sensitivity	Deformation Sensing and Potential Defects Monitoring

## Data Availability

Data are contained within the article.
